# The Natural History of Animals; Being the Substance of Three Courses of Lectures, Delivered before the Royal Institution of Great Britain

**Published:** 1845-04

**Authors:** 


ART. II.—*The Natural History of Animals; being the substance of Three Courses of Lectures, delivered before the Royal Institution of Great Britain.* By THOMAS RYMER JONES, F.R.S., F.Z.S., Professor of Comparative Anatomy in King's College, London. Vol. I. With 105 Illustrations.—*London*, 1845. Small 8vo, pp. 362.

THIS very beautiful little volume appears intended to convey such a general view of the natural history and comparative structure of the different classes of animals, as the intelligent student of Professor Jones's former 'Outline' might present to his female friends; for it consists of essentially the same matter, adapted, both in style and illustration, to be a drawing-room rather than a library book. For this purpose it is admirably designed; its whole getting-up being highly attractive; and its illustrations beyond all praise. Several of them surpass in beauty anything of the kind which we have ever seen; and almost any one of them would have been deemed a marvel of the art of wood engraving, before Mr. Van Voorst commenced the series of illustrated works, which has gained him so high a reputation, and which has done so much to promote the study of natural history. The volume before us includes only the radiated classes, and the lower articulated as high as the myriapoda. Having subjected it to a careful examination, we believe that we may safely pronounce it to be a trustworthy as well as an attractive guide to the study of these branches of natural history,—except in two particulars. 1. We altogether dissent from the notion that the nervous system of the lower animals can exist in the state of "imperceptible molecules diffused throughout the substance of their bodies;" which appears to us to be just as repugnant to the true *idea* of a nervous system, as it would be to that of a circulating system for us to say that it existed in any animals in the condition of minute unconnected cavities diffused through the body, instead of forming a series of continuous channels. 2. We are surprised to find Professor Jones still holding to the doctrine that the *Fungiæ meandrinæ*, and similar zoophytes, are *agastric*, "absorbing from the ocean by means of their whole surface, and not through any mouth, the materials necessary for their sustenance." Distinct proof has been long since given, that these zoophytes have the same general structure with the sea anemone, and are nourished in the same manner. We have ourselves seen and *fed* living *caryophylliæ*; which differ only from fungiæ in being mounted on a footstalk.

